# Lesion region inpainting: an approach for pseudo-healthy image synthesis in intracranial infection imaging

**DOI:** 10.3389/fmicb.2024.1453870

**Published:** 2024-08-19

**Authors:** Xiaojuan Liu, Cong Xiang, Libin Lan, Chuan Li, Hanguang Xiao, Zhi Liu

**Affiliations:** ^1^College of Artificial Intelligence, Chongqing University of Technology, Chongqing, China; ^2^College of Big Data and Intelligent Engineering, Chongqing College of International Business and Economics, Chongqing, China; ^3^College of Computer Science and Engineering, Chongqing University of Technology, Chongqing, China

**Keywords:** pseudo-healthy image synthesis, generative adversarial networks, intracranial infection, data augmentation, contextual residual attention module lesion inpainting for pseudo-healthy synthesis

## Abstract

The synthesis of pseudo-healthy images, involving the generation of healthy counterparts for pathological images, is crucial for data augmentation, clinical disease diagnosis, and understanding pathology-induced changes. Recently, Generative Adversarial Networks (GANs) have shown substantial promise in this domain. However, the heterogeneity of intracranial infection symptoms caused by various infections complicates the model's ability to accurately differentiate between pathological and healthy regions, leading to the loss of critical information in healthy areas and impairing the precise preservation of the subject's identity. Moreover, for images with extensive lesion areas, the pseudo-healthy images generated by these methods often lack distinct organ and tissue structures. To address these challenges, we propose a three-stage method (localization, inpainting, synthesis) that achieves nearly perfect preservation of the subject's identity through precise pseudo-healthy synthesis of the lesion region and its surroundings. The process begins with a Segmentor, which identifies the lesion areas and differentiates them from healthy regions. Subsequently, a Vague-Filler fills the lesion areas to construct a healthy outline, thereby preventing structural loss in cases of extensive lesions. Finally, leveraging this healthy outline, a Generative Adversarial Network integrated with a contextual residual attention module generates a more realistic and clearer image. Our method was validated through extensive experiments across different modalities within the BraTS2021 dataset, achieving a healthiness score of 0.957. The visual quality of the generated images markedly exceeded those produced by competing methods, with enhanced capabilities in repairing large lesion areas. Further testing on the COVID-19-20 dataset showed that our model could effectively partially reconstruct images of other organs.

## 1 Introduction

**Figure 1:**
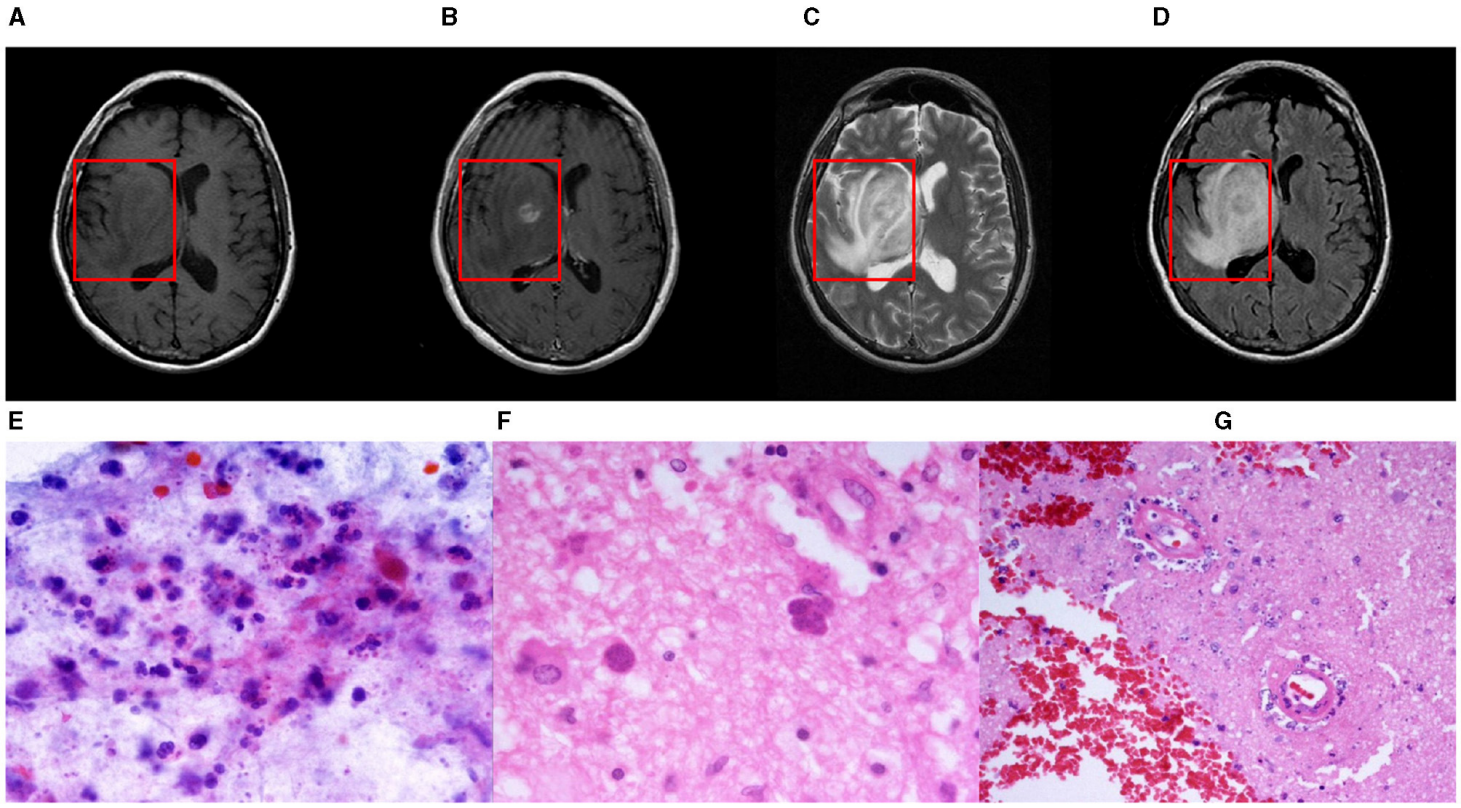
A nodular mass is centered in the right basal nuclei with an irregularly thick contrast-enhancing rim. The central portion of the mass is mildly hypointense to gray matter on T1-weighted imaging and hyperintense on T2-weighted imaging with poor suppression on FLAIR. Histological examination of paraffin sections 1 and 2 confirmed the presence of numerous Toxoplasma gondii tachyzoites and sporadic bradyzoite cysts. These parasites are embedded within a context of extensive cerebral parenchymal necrosis, where a distinct boundary demarcates the necrotic areas from the adjacent viable tissue. Imaging of necrotic regions reveals evidence of necrotic blood vessels, with the accumulation of neutrophils and chronic inflammatory cells, as well as nuclear debris in the surrounding and perivascular regions. Within the surviving parenchyma, reactive proliferation of small blood vessels is observed. Paraffin section 3 displays both cortical and white matter structures. In the deep white matter, there is a significant presence of Toxoplasma gondii tachyzoites along with sporadic bradyzoite cysts situated in areas of congestion and focal necrosis. Sporadic cysts can also be identified in the more superficial white matter and cortical regions. These findings are consistent with a diagnosis of cerebral toxoplasmosis. **(A)** T1-weight, **(B)** T2-weight **(C)** T1ce-weight **(D)** Flair-weight **(E)** Paraffin section 1 **(F)** Paraffin section 2 and **(G)** Paraffin section 3.

Intracranial infections, involving the brain and its adjacent structures, pose significant clinical challenges due to their potential to cause severe outcomes. Characterized by inflammation and infection within the cranial cavity, these conditions affect the brain parenchyma, meninges, and other intracranial structures. A wide range of pathogens, including bacteria, viruses, fungi, and parasites, can instigate various infections such as meningitis, encephalitis, brain abscesses, and subdural empyemas (Foerster et al., 2007). Magnetic Resonance Imaging (MRI) is crucial in detecting, assessing, and monitoring these central nervous system infections and inflammations (Zimmerman and Girard, 2004; Mitchell and Dehkharghani, 2014). By providing comprehensive imaging of the brain and its meningeal coverings, MRI helps identify distinct patterns and features indicative of different types of intracranial infections. For example, MRI excels in distinguishing between pyogenic and fungal abscesses; pyogenic abscesses typically present with a well-defined rim and surrounding edema. In cases of ventriculitis (Luque-Paz et al., 2021), MRI can display ventricular enlargement, ependymal enhancement, and intraventricular debris. Enhancing MRI images in instances of intracranial infection is thus essential, as it provides clinicians with critical diagnostic information, improving both diagnostic accuracy and efficiency. Figure 1 illustrates an instance of intracranial infection (Deng and Gaillard, 2014).

In recent years, the development of pseudo-healthy image synthesis technology has become a pivotal tool in data augmentation and medical image anomaly detection. In the realm of data augmentation, generating pseudo-healthy images significantly enriches datasets by creating numerous representations from the same subjects' pathological images (Xia et al., 2020). This technique not only bolsters the model's generalization capabilities but also mitigates challenges associated with data imbalance and limited sample availability. In anomaly detection within medical imaging, synthesizing pseudo-healthy images allows models to simulate representations of healthy tissues (Tsunoda et al., 2014). By contrasting these images with their pathological counterparts, clinicians can more accurately pinpoint lesions. Thus, the production of high-quality pseudo-healthy images is crucial for enhancing the detection and diagnosis of conditions like intracranial infections. Furthermore, comparing pathological images with pseudo-healthy ones deepens the understanding of pathology-induced alterations, thereby advancing insights into disease progression and pathology development processes.

The process of synthesizing pseudo-healthy images involves generating apparently normal, lesion-free images from pathological data using sophisticated computer imaging and machine learning techniques. Ideally, a pseudo-healthy image should possess two essential attributes (Zhang et al., 2022): First, the image must maintain a healthy appearance, closely mimicking a genuine healthy image. This is the primary goal of pseudo-healthy image synthesis. Second, the synthesized image must originate from the same individual as the pathological image. This requirement is equally important, as producing healthy images from different individuals does not aid in medical diagnosis (Bowles et al., 2016). Typically, it is not feasible for the tissues or organs of a single patient to exhibit both pathological and healthy states simultaneously. Therefore, identifying an exact corresponding pseudo-healthy image for a specific pathological image is inherently complex and fraught with uncertainties. In the context of pseudo-healthy synthesis for intracranial infection, the varied manifestations of the disease in MRI images present significant challenges. For instance, severe cerebral edema in lesion areas can cause a mass effect, compressing and deforming adjacent brain ventricles. Consequently, pseudo-healthy synthesis for intracranial infection should focus on restoring the anatomical integrity in affected regions and accommodating the disease's diverse presentations.

The synthesis of pseudo-healthy images entails creating seemingly normal, lesion-free images from pathological data through the use of advanced computer image processing and machine learning techniques. Determining whether an image is truly pseudo-healthy hinges on the absence of pathological features, while maintaining the subject's identity depends on the intact preservation of non-pathological regions. Consequently, in pseudo-healthy synthesis, accurately localizing pathological regions and reconstructing their healthy analogs is paramount. Several Generative Adversarial Network (GAN)-based approaches for pseudo-healthy image synthesis have been previously proposed (Baumgartner et al., 2018; Chen and Konukoglu, 2018; Baur et al., 2019, 2020). These methods typically employ a generator, structured as an encoder-decoder network, to convert pathological images into their healthy-looking equivalents. Simultaneously, a discriminator, competing against the generator, utilizes a classifier to differentiate between the synthesized healthy images and actual healthy images. Through this adversarial training process, the generator and classifier refine their capabilities in a dynamic interplay. However, a significant limitation of these methods is their inability to effectively learn and incorporate pathological information, which complicates the task of maintaining the subject's identity in the synthesized pseudo-healthy images. To overcome these challenges, Xia et al. (2020) and Zhang et al. (2022) introduced the use of a segmentor alongside pixel-level annotations. This strategy involves the collaborative training of both the generator and the segmentor. The segmentor's training loss is fed back to the generator, encouraging it to differentiate pathological information from the subject's identity while preserving any healthy attributes present in the pathological image. Despite these advancements, the methods still face several drawbacks.

(1) Integrating the segmentor into the model results in an overdependence on the segmentor's efficacy for lesion localization. The varied etiologies underlying intracranial infections lead to significantly diverse symptoms. Thus, a singular segmentation strategy is evidently inadequate to meet these demands.

(2) The generator creates segments devoid of lesion regions, whereas the classifier's visual focus is primarily on healthy areas. This causes the generator to employ images from different subjects to deceive the discriminator, unintentionally erasing the unique identity of the subject.

(3) These models demonstrate a deficiency in learning anatomical structures from healthy images, thus hindering their capacity to accurately reconstruct anatomical features within lesion areas, especially in cases involving extensive lesions.

**Figure 2:**
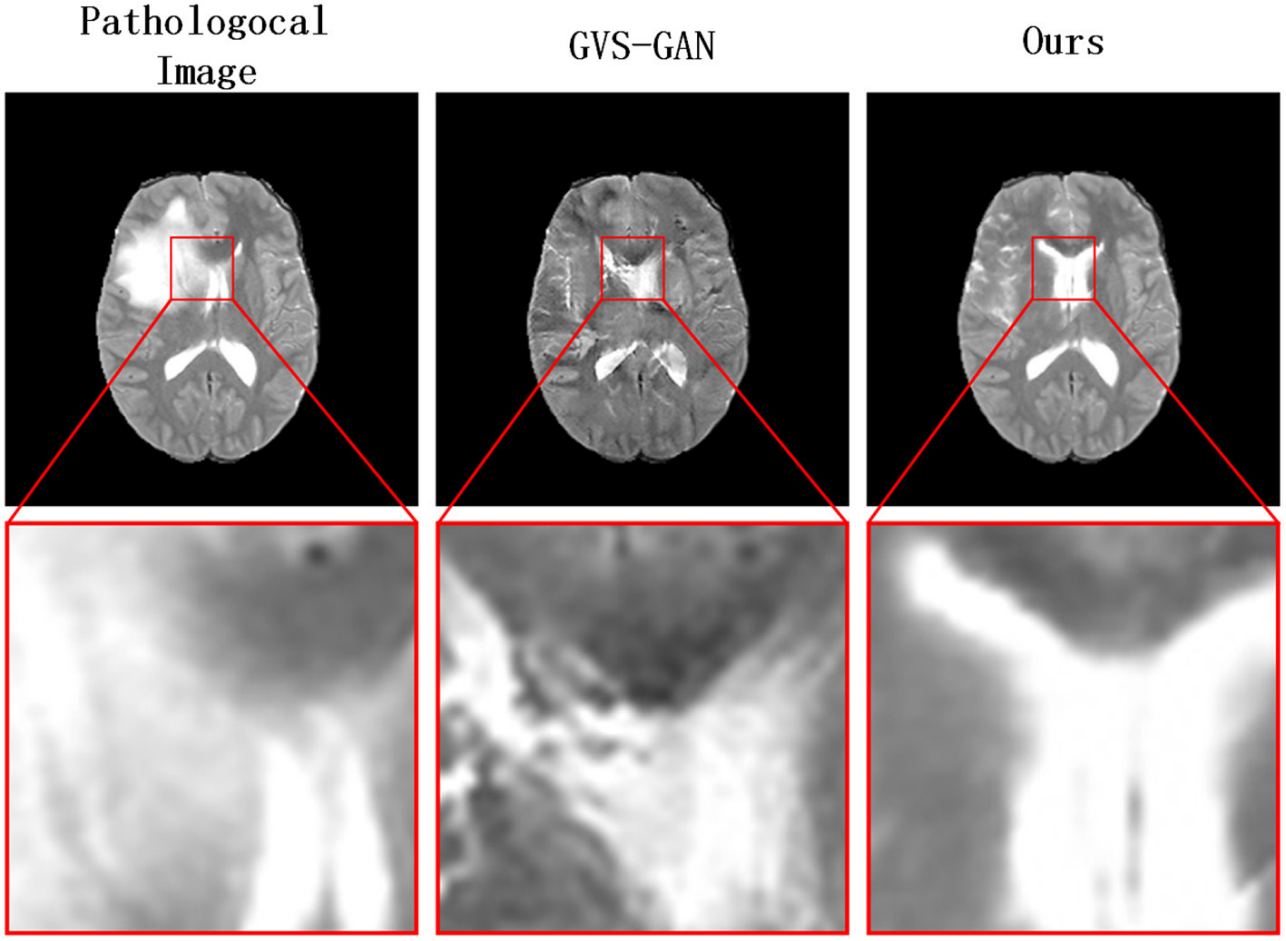
Our model generates clearer pseudo-healthy images in the presence of large lesions compared to the current state-of-the-art model GVS-GAN.

To address the challenges and accommodate the anatomical alterations caused by intracranial infections, we introduce a novel three-stage pseudo-healthy image synthesis model called the Lesion Region Inpainting Generative Adversarial Network (LRI-GAN). This model is specifically tailored to manage the varying characteristics of infection areas in brain imaging. It ensures the preservation of the subject's identity by accurately synthesizing and replacing lesioned areas with pseudo-healthy regions, thus maintaining image integrity. The three-stage architecture enhances the model's effectiveness in constructing accurate healthy contours. Initially, various segmentation models are pre-trained, based on specific pathological requirements, or employing pixel-level annotations from clinical experts to precisely pinpoint lesion areas. Subsequently, in the second stage, a “Vague-filler” network fills the identified lesion regions, including an adjacent 5 mm area, capturing the essential characteristics of healthy tissues. The final stage employs a Generator network, enhanced with a contextual residual attention module, which adeptly learns from real healthy images and extracts relevant features from non-lesioned parts of the pathological image. This innovative approach results in pseudo-healthy images that not only reflect a clearer visual quality but also enhance diagnostic accuracy, as demonstrated in Figure 2. The LRI-GAN thus represents a significant advancement in medical imaging, particularly in the synthesis of images for diagnostic and treatment planning in cases of intracranial infection.

To assess the efficacy of our proposed method, we utilized image slices from the BraTS2021 dataset, featuring various conditions like edema, hemorrhage, and deformation. Our extensive testing shows that this method surpasses contemporary leading techniques in performance. Further validation was conducted using the COVID-19-20 dataset to evaluate the model's versatility across different organs, confirming consistent high performance.

Key contributions of our study include:

(1) Development of an advanced pseudo-healthy image synthesis approach tailored for intracranial infections, which preserves the identity of the pathological region with meticulous lesion area restoration.

(2) Introduction of a novel generator network architecture, incorporating a flipped symmetrical structure and a contextual residual attention mechanism, designed specifically to accurately mend lesioned areas.

(3) Establishment of a new evaluation metric called “Structure Healthiness” (SH), designed to gauge the capability of models to restore the anatomical integrity of lesion areas.

## 2 Related works

In the field of medical image analysis, the synthesis of pseudo-healthy images has attracted significant interest due to its potential benefits for various downstream applications. Research in this area can be categorized into two main groups based on the nature of the training data utilized (Zhang et al., 2022). The first category is Pathology-deficiency based methods. These methods exclusively rely on healthy images during the training process and are consequently devoid of pathological information. They do not require pathological data for training and are often closely associated with unsupervised medical image segmentation techniques (Bowles et al., 2017; Baumgartner et al., 2018; Tao et al., 2023; Rahman Siddiquee et al., 2024). The second category comprises Pathology-sufficiency based methods, which utilize a comprehensive dataset containing both pathological and healthy images during training. These approaches address the challenge of pseudo-healthy image synthesis from an image translation perspective. They incorporate pathological images along with their corresponding image-level or pixel-level pathological annotations to ensure that the synthesized pseudo-healthy images closely resemble the characteristics of healthy tissues (Sun et al., 2020; Xia et al., 2020; Zhang et al., 2022). This methodology facilitates more accurate and clinically relevant outputs by incorporating essential pathological details into the training process.

### 2.1 Pathology-deficiency based methods

Pathology-deficiency based methods begin by learning the normative distribution, leveraging techniques focused on compressing and recovering structures of healthy anatomical features during training. Subsequently, during the testing phase, these methods compress pathological images into a latent space. The underlying hypothesis is that the resultant latent representations closely approximate those of pseudo-healthy images, leading to the reconstruction of pseudo-healthy images from these representations. Chen and Konukoglu (2018) utilized an autoencoder-based approach to capture the distribution of brain MRIs from healthy subjects. Their objective was to map images to regions proximate to corresponding healthy images in latent space, employing specific constraints to guide this process. In a similar vein, Baur et al. (2019) modeled the distribution of healthy brain MRIs to identify pathological alterations through erroneous reconstructions. They implemented a Laplacian pyramid technique to compress and reconstruct healthy brain MRIs, which resulted in higher reconstruction fidelity at greater resolutions. Nevertheless, such methods are founded on idealized assumptions that often do not hold in practical scenarios. Specifically, the challenge lies in identifying an optimal latent representation that aligns with pseudo-healthy images when pathological images are compressed into the latent space. This difficulty frequently leads to a failure to preserve the identity of the pseudo-healthy images. Therefore, while the theoretical foundation of these methods is strong, their practical application is hindered by limitations in capturing and maintaining the true characteristics of the subject's healthy state in the synthesized images.

### 2.2 Pathology-sufficiency based methods

To synthesize higher-quality pseudo-healthy images, VA-GAN (Baumgartner et al., 2018) introduces a GAN-based framework that incorporates pathological information. This framework comprises a generator, tasked with synthesizing images that appear healthy while preserving the subject's identity, and a discriminator, which distinguishes between these synthesized images and real, unpaired healthy images. However, this method relies primarily on image-level annotations, which limits its ability to accurately differentiate between lesioned and non-lesioned areas, consequently impacting the preservation of the subject's identity in the synthesized images. To mitigate these limitations, PHS-GAN (Xia et al., 2020) and ANT-GAN (Sun et al., 2020), both variants of Cycle-GAN, incorporate pixel-level annotations. PHS-GAN addresses the one-to-many issue characteristic of medical images with variable pathology by employing a segmenter alongside pixel-level pathological annotations. This configuration allows precise localization of lesions, facilitating the separation of pathological information from healthy tissue, thus enhancing the precision of pseudo-healthy image synthesis. This method effectively manages pathological data to improve the accuracy and realism of the generated images. ANT-GAN, on the other hand, utilizes the L2 loss calculated between non-lesioned areas of the pathological and pseudo-healthy images. By reintegrating this feedback into the entire cyclic network, ANT-GAN ensures that the identity of the subject is maintained in the resultant images. To improve the localization of lesions, GVS-GAN (Zhang et al., 2022) attempts to resolve discrepancies between how healthy and pseudo-healthy images are perceived by the segmenter, aiming for a harmonized outcome. Nonetheless, these strategies, by trying to make the segmenter less sensitive to lesions, may not truly achieve the creation of “pseudo-healthy” images in the strictest sense. A persistent challenge with these methods is their struggle to fully grasp the anatomical features of a healthy brain, especially when faced with images featuring extensive lesions. This often leads to the generated images obscuring rather than restoring the anatomical structure of the affected areas. Consequently, while these approaches advance the field of pseudo-healthy image synthesis by better managing pathological information and improving image realism, they still face significant hurdles in accurately rendering and restoring the detailed anatomy in areas affected by pathology.

### 2.3 Our method

To facilitate the synthesis of pseudo-healthy images for intracranial infections, we have integrated the aforementioned methods and introduced a segmentation-first, then-repair strategy for pseudo-healthy synthesis. This approach differs from previous methodologies, which incorporated the segmentor within the generative network during the training phase, thus performing segmentation and generation simultaneously. Instead, our method employs the segmentor specifically to localize lesion areas, a strategy that prevents the segmentor from excessively influencing the generative network during training and ensures that the generator does not focus disproportionately on concealing lesions. Additionally, this segmentation-first approach allows for the flexible replacement of the segmentor, enhancing the model's adaptability to the varied manifestations of intracranial infections evident in MRI images. Our method executes the synthesis of pseudo-healthy images in a structured three-stage process. Initially, in the first stage, lesion areas are precisely identified using either a pre-trained segmentor model or manual annotations. Following this, the second stage employs a Vague-Filler network designed to infill these localized lesion areas, effectively mimicking the appearance of healthy tissue. In the final stage, a generator equipped with an inverted symmetrical structure and a contextual residual attention module (Yi et al., 2020) is utilized. This sophisticated arrangement enables the generator to learn effectively from both flipped images and features outside the lesion areas, thereby enhancing its capability to synthesize more accurate pseudo-healthy images tailored to the specific requirements of intracranial infection cases.

## 3 Methods

The architecture of LRI-GAN comprises three distinct components aligned with the workflow: a Segmentor (responsible for localization), a Vague-Filler (responsible for coarse filling), and a Generator (responsible for fine reconstruction). Both the Vague-Filler and the Generator are trainable elements, whereas the Segmentor is a pre-trained deep learning model or manually annotated pixel-level pathology. The structure of this paper is as follows: Section 3.1 provides an overview of the problem; Section 3.2 introduces the Segmentor; Section 3.3 describes the Vague-Filler; Section 3.4 elaborates on the Generator; Section 3.5 discusses the loss function of LRI-GAN; Section 3.6 outlines the training process of LRI-GAN; and Section 3.7 details the inference procedure of LRI-GAN.

### 3.1 Problem overview

**Figure 3:**
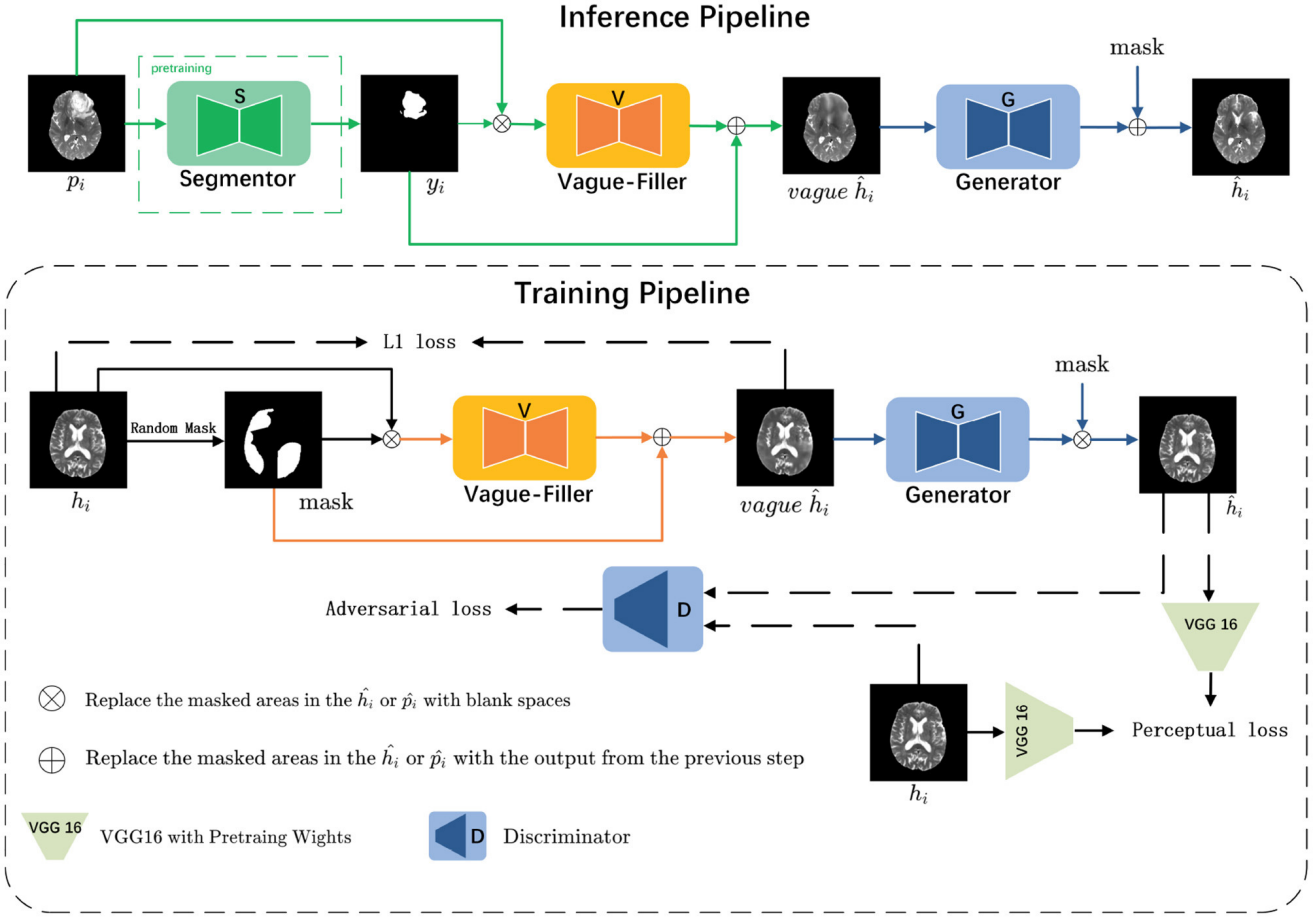
The LRI-GAN mainly consists of three components: the Segmentor, the Vague-Filler, and the Generator. During the training stage, the model is trained on healthy images. In the inference stage, it is applied to pathological images.

As illustrated in Figure 3, we consider a set of images ${\left\{x_{i}\right\}}_{i=1}^{N}\in X$, with each *i* representing a slice, alongside their binary annotations ${\left\{y_{i}\right\}}_{i=1}^{N}\in Y$. These images are classified into two subsets based on their labels: pathological images ${\left\{p_{i}\right\}}_{i=1}^{M}$ and healthy images ${\left\{h_{i}\right\}}_{i=1}^{N-M}$. The data distributions of the pathological and healthy samples are denoted as *p*_*i*_~*f*_*p*_ and *h*_*i*_~*f*_*h*_, respectively.

In the inference pipeline, for a given pathological image *p*_*i*_ that contains lesion regions, our objective is to derive the corresponding *y*_*i*_ (where 0 indicates normal regions and 1 indicates pathological regions) via the Segmentor S. Subsequently, *y*_*i*_ is combined with *p*_*i*_ and fed into the Vague-filler V to produce a vague pseudo-healthy image *vagueĥ*_*i*_. This image is then refined by the Generator G to yield a clearer pseudo-healthy image ĥ_*i*_, ensuring that ĥ_*i*_ adheres to the distribution of healthy images (i.e., ĥ_*i*_~*f*_*h*_). Moreover, we aim to maintain the normal anatomical structure of *p*_*i*_ within ĥ_*i*_.

In the training pipeline, we emphasize healthy images to comprehensively learn their latent features. For a given healthy image *h*_*i*_, we randomly mask 30%–60% of the regions to emulate the process of a pre-trained Segmentor detecting lesion regions, resulting in the corresponding mask *y*_*i*_. This mask is then combined with *h*_*i*_ and input into the Vague-filler V to generate a vague pseudo-healthy image *vagueĥ*_*i*_. The Generator G is then utilized to refine *vagueĥ*_*i*_, producing a clearer pseudo-healthy image ĥ_*i*_, which ensures that the masked regions in ĥ_*i*_ closely resemble the original healthy image *h*_*i*_.

### 3.2 Segmentor

Before commencing the synthesis of pseudo-healthy images, accurately identifying lesion locations within pathological images is crucial. The primary aim during the Segmentor phase is to obtain pixel-level annotations *y*_*i*_ that precisely delineate lesion areas in the pathological image *p*_*i*_. However, acquiring such detailed pathological annotations is often expensive and time-consuming. Therefore, for pathological images lacking specific annotations, we utilize a pre-trained segmentor, S, to automatically generate these annotations. In this study, we employ the U-Net architecture, renowned for its effectiveness in medical image segmentation, as the pre-trained segmentor (Ronneberger et al., 2015).

### 3.3 Vague-Filler

**Figure 4:**
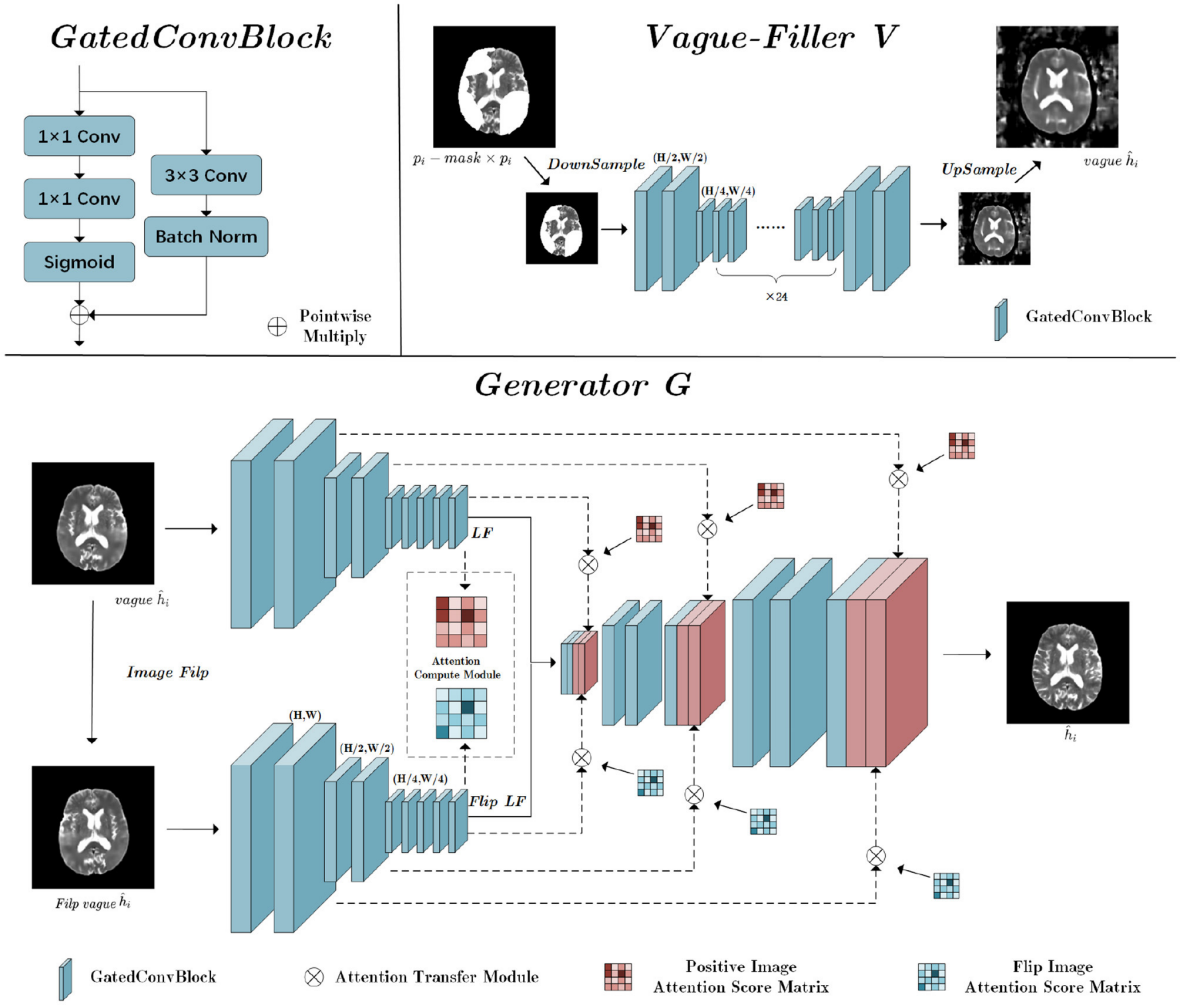
The structure of Vague-Filler V and Generator G; H is the height of the input image, and W is the width of the input image.

The Vague-Filler processes the pathological image *p*_*i*_, where lesion regions are replaced by blanks, to produce a preliminary pseudo-healthy image, *vagueĥ*_*i*_. Detailed insights into the Vague-Filler's methodology are provided in the “Vague Filler” section illustrated in Figure 4. This component accepts an image alongside a binary mask of lesion regions as inputs and outputs a filled-in image. It incorporates gated convolution as its sole learnable mechanism. The Vague-Filler operates on a “straight-line” residual network architecture devoid of skip connections, preserving the input and output dimensions at H × W pixels. To broaden the receptive field and minimize computational demands, the input image is initially down-sampled to $\frac{H}{2}\times \frac{W}{2}$ pixels prior to convolution. Subsequent convolutions further reduce the resolution to $\frac{H}{4}\times \frac{W}{4}$ pixels using two gated convolutions. The image then undergoes additional processing at the $\frac{H}{4}\times \frac{W}{4}$ scale via a sequence of gated convolutions, which vary in stride and padding, yet maintain a consistent size throughout the input and output stages.

### 3.4 Generator

The Generator's fundamental role is to enhance a vaguely defined pseudo-healthy image, denoted as *vagueĥ*_*i*_, into a distinctly clearer image ĥ_*i*_. This enhancement recognizes the symmetric nature of brain medical imagery, incorporating a flip-symmetric architecture detailed in the Generator section of Figure 3. Initially, *vagueĥ*_*i*_ undergoes a flipping operation to prepare for convolutional processing. Both the original and flipped versions of *vagueĥ*_*i*_ undergo parallel convolutional operations.


(1) $$\begin{array}{l} flip\;vague{\hat{h}}_{i}=flip\left(vague\;{\hat{h}}_{i}\right) \end{array}$$

The convolution phase features a fully symmetric dual-path structure that optimizes feature extraction:


(2) $$\begin{array}{l} \text{LF}=\text{down\_conv}\left(\text{vague}\;{\hat{h}}_{i}\right) \end{array}$$

(3) $$\begin{array}{l} \text{LF\_flip}=\text{down\_conv}\left(flip\;vague{\hat{h}}_{i}\right) \end{array}$$

*LF* represents the latent features derived post-convolution, and *down_conv* refers to the down-sampling convolution process. The Attention Calculation Module (ACM) calculates attention score matrices for both the forward and flipped images:


(4) $$\begin{array}{l} PIAS\;Matrix=ACM\left(LF\right) \end{array}$$

(5) $$\begin{array}{l} FIAS\;Matrix=ACM\left(LF\text{\_}flip\right) \end{array}$$

The PIAS Matrix denotes the Positive Image Attention Score Matrix, detailing the interactions of the forward image with the mask area, while the FIAS Matrix is the Flip Image Attention Score Matrix, detailing interactions of the flipped image components with the mask. After calculating these matrices, both pathways integrate the residuals within the masked areas using their respective Attention Transfer Modules (ATM), based on the attention scores and contextual residuals:


(6) $$\begin{array}{l} {\hat{h}}_{i}=conv\left(LF,LF\text{\_}flip,ATM\left(LF,PIAS\;Matrix\right),ATM\left(LF\text{\_}flip,FIAS\;Matrix\right)\right) \end{array}$$

The ACM uses cosine similarity measures for establishing image attention scores across high-level feature maps:


(7) $$\begin{array}{l} c_{i,j}=\langle \frac{b_{i}}{||b_{i}||},\frac{b_{j}}{||b_{j}||}\rangle \end{array}$$

*b*_*i*_ and *b*_*j*_ represent the patches outside and inside the mask area, respectively. The resultant similarity scores are squared and normalized to derive attention scores for each patch:


(8) $$\begin{array}{l} s_{i,j}=\frac{c_{i,j}^{2}}{\sum_{i=1}^{N}c_{i,j}^{2}} \end{array}$$

N represents the number of patches outside the mask area. Despite the heterogeneity in lesion areas, a 256–256 matrix uniformly stores potential affinity scores between any pair of patches.

Finally, the ATM utilizes these attention scores to fill gaps in the low-level feature map with contextually weighted patches:


(9) $$\begin{array}{l} b_{j}=\overset{N}{\underset{i=1}{\sum }}s_{i,j}b_{i} \end{array}$$

*b*_*i*_ is extracted from outside the masked area and *b*_*j*_ fills within the mask. Each patch measures 16 × 16, allowing for the extraction of 256 patches in total.

Through residual aggregation, the model reconstructs detailed aspects of the lesion area:


(10) $$\begin{array}{l} R_{j}=\overset{N}{\underset{i=1}{\sum }}s_{i,j}R_{i} \end{array}$$

*R* denotes the residual image, with *R*_*i*_ and *R*_*j*_ representing the patches involved in filling the masked area. These patches cover all pixels seamlessly, ensuring a coherent integration of filled residuals with the surrounding tissue. The resultant aggregated residual image is then merged with the up-sampled blurry image from the generator to enhance clarity.

### 3.5 Loss function

#### 3.5.1 Vague-Filler loss

**L1 Loss:** To ensure uniformity throughout the training process of the Vague-Filler, we utilize the L1 loss function. The formula for this is given by:


$$L_{v}=\frac{1}{N}\overset{N}{\underset{i=1}{\sum }}|h_{i}-\text{vague}{\hat{h}}_{i}|$$

In training the Vague-Filler, our objective is to enhance the model's focus on the contour structures of healthy images, while allowing a greater tolerance toward their textural features. The L1 loss function is chosen because it minimally penalizes large discrepancies and accommodates outliers effectively, making it an appropriate choice for this application.

#### 3.5.2 Generator loss

To enhance the stability of the generator's training, we use the hinge loss method for adversarial training. Additionally, to enrich texture details in the generated images, we incorporate perceptual loss.

**Adversarial loss:** For the adversarial training of the generator, we employ the hinge loss method. The primary goal is to maximize the separation between positive and negative samples, thus enhancing categorical distinctions. This approach is based on the methodology used in the Geometric GAN (Lim and Ye, 2017), which has demonstrated improvements in the effectiveness of adversarial training. The adversarial losses for the discriminator and the generator are defined as follows:


$$L_{D}=E\left[\max\left(0,1-D\left(h_{i}\right)\right)\right]+E\left[\max\left(0,1+D\left(G\left(p_{i}\right)\right)\right)\right]$$

$$L_{G}=-E\left[D\left(G\left(h_{i}\right)\right)\right]$$

Here, *G* represents the generator, and *D* represents the discriminator. For *D*, only positive samples where *D*(*X*) < 1 and negative samples where *D*(*G*(*z*))>−1 impact the outcome, implying that a small fraction of samples exceeding these thresholds will not influence the gradients. This results in more stable training dynamics.

**Perceptual loss:**To ensure that the images generated by the generator network during high-definition reconstruction closely align with the visual characteristics of healthy tissues, we incorporate perceptual loss. Perceptual loss emphasizes the perceptual quality of the restored images rather than solely focusing on pixel-level differences. This loss is widely used in medical imaging to enhance the restoration of textural details (Yang et al., 2018; Li et al., 2021). The perceptual loss is defined as follows:


$$L_{perc}\left(G\right)=\lambda E\left[\left||\phi \left(h_{i}\right)-\phi \left(G\left(h_{i}\right)\right)|\right|\right]$$

Here, ϕ denotes the feature extraction function from the VGG16 network, and λ, the weight of the texture loss, is set to 64. The final loss function of the generator is:


$$\text{loss}=L_{G}+L_{perc}\left(G\right)$$

### 3.6 Training pipeline

The training process is elucidated within the “Training Pipeline” section, as depicted in Figure 3. During this phase, the grayscale values of all images are linearly adjusted to a range of [−1, 1]. Masks are designated by a value of 1 for missing regions and 0 for background areas. In this context, *h*_*i*_ represents the input healthy image, ${\tilde{h}}_{i}$ denotes the generated healthy image, and *m* indicates the mask for missing regions. The operation ⊙ signifies element-wise multiplication. The Vague-Filler *V* interprets the concatenated masked image and mask as inputs to forecast the vague image vague_*y*_ = *V*(*h*_*i*_, *m*), maintaining the same dimensions as the input image. Following this, the Generator *G* uses the combined vague image and mask to predict *y* = *G*(vague_*y*_, *m*), producing a pseudo-healthy image with dimensions identical to those of the input image. Detailed descriptions of this training process are provided in Algorithm 1.

**Algorithm 1:**
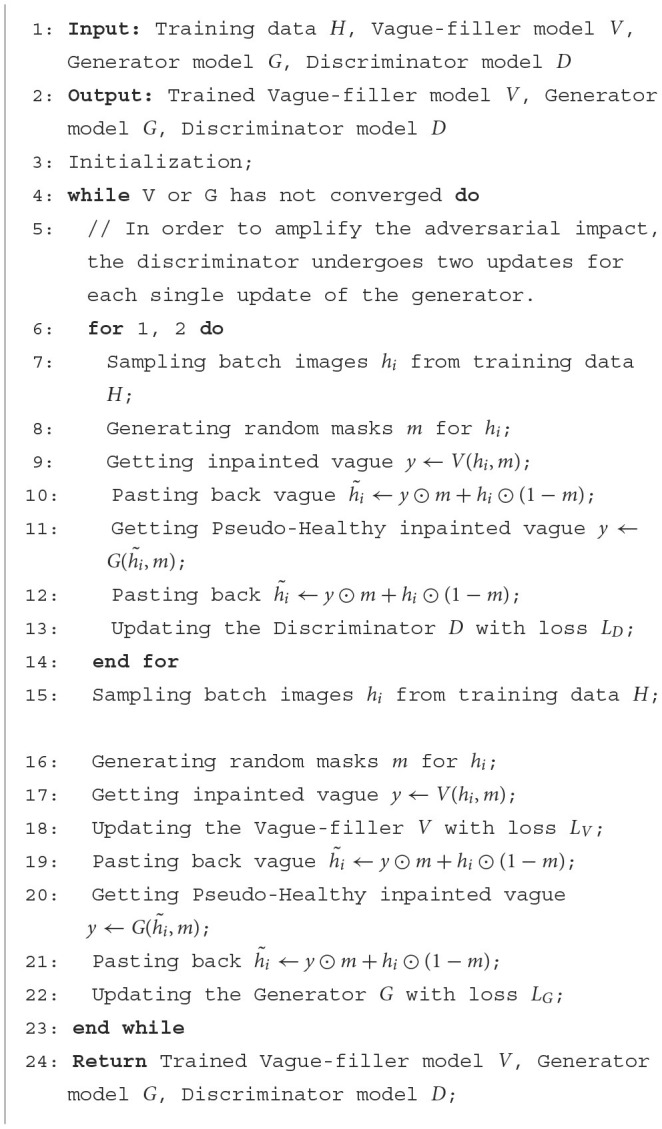
Training of our approach.

### 3.7 Inference pipeline

The inference process is detailed within the “Inference Pipeline” section, as outlined in Figure 3. During inference, the grayscale values of all images are linearly adjusted to range from [−1, 1]. Masks are used to indicate pathological regions with a value of 1 and background areas with a value of 0. In this context, *p*_*i*_ represents the input pathological image, while ${\tilde{h}}_{i}$ signifies the generated healthy image post vague filling. The operation ⊙ stands for element-wise multiplication. The Vague-Filler *V* processes the concatenated masked image and mask as input, forecasting a vague filled image vague*y* = *V*(*h*_*i*_, *m*) that retains the dimensions of the input image. Subsequently, the Generator *G* utilizes the combined vague image and mask to generate $y=G\left(\text{vague}\left({\tilde{h}}_{i}\right),m\right)$, resulting in the pseudo-healthy image ${\tilde{h}}_{i}$. This process is comprehensively described in Algorithm 2.

**Algorithm 2:**
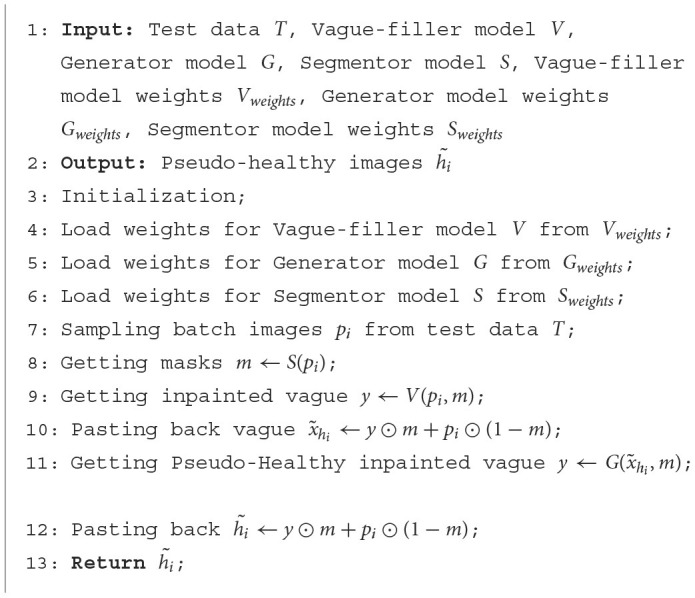
Inferencing of our approach.

## 4 Experiments

### 4.1 Datasets

The proposed model was rigorously evaluated using the T1 and T2 modalities of the BraTS2021 dataset, demonstrating effectiveness across the T1ce and FLAIR modalities as well. The model's versatility was further assessed by examining its adaptability to viral lesions in other organs with the COVID-19-20 dataset.

BraTS2021 Dataset (Menze et al., 2015; Bakas et al., 2017; Baid et al., 2021): The BraTS2021 Dataset (Brain Tumor Segmentation Challenge 2021 Dataset) is designed for the task of medical image segmentation, specifically aimed at evaluating and advancing algorithms for brain tumor segmentation. It comprises MRI scans of the brain collected from multiple medical centers. Each case in the dataset includes four different MRI modalities: T1-weighted, T2-weighted, T1-weighted with contrast enhancement (T1ce), and Fluid-Attenuated Inversion Recovery (FLAIR), along with corresponding ground truth tumor segmentation. Comprising 1,251 cases in the training set, 219 in the validation set, and 530 in the test set, the BraTS2021 dataset ensures comprehensive evaluation. All cases are skull-stripped, resampled to an isotropic resolution of 1 mm^*s*^, and co-registered. Each volume presents four modalities: T1, T2, T1ce, and FLAIR, measured at dimensions of 240 × 240 × 155 (L × W × H).

COVID-19-20 Dataset (Roth et al., 2022): The COVID-19-20 challenge facilitates the evaluation of innovative techniques for segmenting and quantifying lung lesions induced by SARS-CoV-2 through CT images. Drawn from multiple institutions across various countries, these images depict a diverse cohort in terms of age, gender, and disease severity. The dataset includes 199 training images and 50 validation images, each with a resolution of 512–512 pixels. Notably, these images detail lung lesions caused by SARS-CoV-2 and include ground truth annotations derived from non-contrast chest CT scans with confirmed positive RT-PCR results.

### 4.2 Implementation details and baseline comparisons

**Environment:** Windows 11, CUDA 11.7.

**Framework**: The methodology is implemented using the PyTorch framework.

**Optimizer**: Model training is facilitated using the Adam optimizer.

**Learning rate**: The initial learning rate is set at 0.001 and reduces by 50% every 5 epochs.

**Batch size**: Given the slice dimensions of the BraTS2021 dataset at 240 × 240 and those of the COVID-19-20 dataset at 512 × 512, batch sizes are accordingly adjusted. A batch size of 16 is employed for the BraTS2021 dataset, while a smaller batch size of 4 is utilized for the COVID-19-20 dataset.

**Training hardware**: The model is trained on an NVIDIA GeForce 4080 Super 16GB GPU.

**Compared methods**: The effectiveness of the proposed method is assessed against three pathologically-informed pseudo-healthy synthesis approaches [GVS-GAN (Zhang et al., 2022), PHS-GAN (Xia et al., 2020), and VA-GAN (Baumgartner et al., 2018)] and two widely-used generative adversarial models [AAE (Makhzani et al., 2016) and Cycle GAN (Zhu et al., 2020)].

**Code sources**: For the implementation, official codebases are used for GVS-GAN, VA-GAN, and PHS-GAN, while the most popular GitHub repositories are sourced for AAE and Cycle GAN.

**Data processing:** For the BraTS2021 dataset, we extracted one slice every five slices, resulting in a total of 13,759 slices. For the COVID-19-20 dataset, we filtered the slices to include only those with clearly visible lungs, extracting one slice every two slices, which yielded a total of 2,965 slices.

### 4.3 Structure healthiness

**Figure 5:**
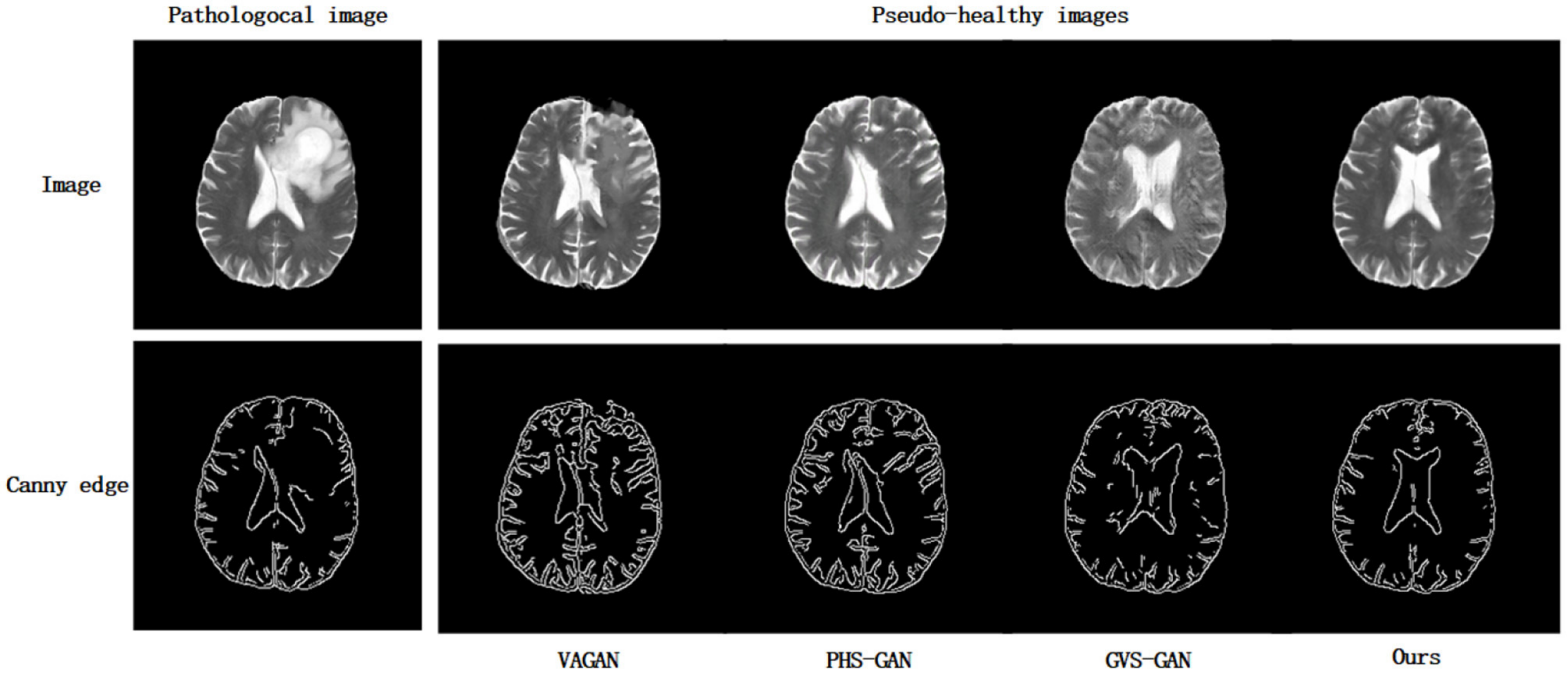
The pseudo-healthy synthetic images generated by VAGAN, PHS-GAN, GVS-GAN, and the method proposed in this paper, alongside their corresponding edge maps that display the anatomical structures.

In certain instances, significant deformations are often observed in pathological images, particularly when large lesion areas are present. Figure 5 illustrates pseudo-healthy images and their corresponding Canny edge maps synthesized under such conditions. Notably, it is common for models to still generate pseudo-healthy images with deformations. To address this issue, Xia et al. (2020) suggested the use of a classifier to categorize Canny edge maps of both healthy and lesioned images to evaluate the presence of deformations. Despite this approach, our statistical analysis of 13,759 pathological slices revealed that only 1,059 slices presented large lesions, where the lesion area exceeded 20% of the total brain area. This indicates that large lesions are relatively rare among pathological slices. Therefore, solely classifying Canny edge maps of healthy and lesioned images does not provide a reliable assessment of a model's deformation correction capability in cases with extensive lesions.

Building on the methodology, we introduce the concept of “Structural Health” (SH) to more accurately explore models' abilities to correct deformations in images with substantial lesion areas. We specifically employed the BraTS21 dataset for this purpose, analyzing Canny edge maps of medical images both with and without extensive lesions. A binary classifier, trained on the VGG network, was utilized. This classifier demonstrated a high level of performance, achieving an average accuracy of 91.2% during its pre-training phase, which underscores its efficacy in detecting deformations in images. The classifier's output, a continuous value ranging from 0 to 1, indicates the likelihood of an image being free from deformations. During the evaluation phase, we focused exclusively on pseudo-healthy images generated from samples with extensive lesions. Here, SH is quantified as the average probability that these images maintain structural integrity and are free from deformations.


(11) $$\begin{array}{l} SH=E_{x_{p}\sim P}\left[C_{p}\left(GeN\left(x_{p}\right)\right)\right] \end{array}$$

In this formula, *x*_*p*_ denotes the pathological image, *C*_*p*_ represents the pre-trained edge map classification model, and GeN indicates the pseudo-healthy synthesis network.

### 4.4 Other metrics

#### 4.4.1 Healthiness

To evaluate the “healthiness” of pseudo-healthy images, Xia et al. (2020) developed a metric named “healthiness.” This metric utilizes a pre-trained segmentation model, which is further refined on a validation set. The fundamental role of this segmenter is to identify pathological regions within both generated pseudo-healthy images and their original pathological counterparts. The healthiness metric is quantified by the proportion of matching pathological pixels found in these images, where a higher percentage indicates a more extensive presence of pathological regions, thus denoting a lower healthiness. The healthiness index (HEALTHINESS, *H*) is calculated using the following formula:


$$H=1-\frac{E_{x_{p}\sim P}\left[N\left(f_{p}\left(\text{GeN}\left(x_{p}\right)\right)\right)\right]}{E_{m_{p}\sim p_{m}}\left[N\left(f_{p}\left(x_{p}\right)\right)\right]}$$

Here, *x*_*p*_ represents the pathological image, *f*_*p*_ is the pre-trained segmentation model, *N*(·) denotes the number of pixels identified as pathological by *f*_*p*_, and GeN refers to the pseudo-healthy generation network. The denominator incorporates the segmentation output *f*_*p*_(*x*_*p*_) of the pathological image rather than the actual mask *m*_*p*_, to counter potential biases from the pre-trained model. Subtracting this term from 1 indicates that a reduction in the pathological mask correlates with an increase in *H*, signifying enhanced healthiness.

#### 4.4.2 Identity preservation

The metric for Identity Preservation quantifies the degree to which the generated pseudo-healthy images maintain the subject's identity (Zhang et al., 2022), specifically assessing the likelihood that both the synthesized pseudo-healthy image and the input pathological image are derived from the same subject. This metric evaluates the structural similarity and peak signal-to-noise ratio of non-pathological regions between the pseudo-healthy image and its corresponding pathological counterpart. The calculations are based on the following formulas:


(12) $$MP=PSNR[(1-y_{t})⊙G(x_{p}),(1-y_{t})⊙x_{p}]$$

(13) $$MS=SSIM[(1-y_{t})⊙G(x_{p}),(1-y_{t})⊙x_{p}]$$

Where *x*_*p*_ represents the pathological image, *y*_*t*_ denotes the corresponding pathological mask, ⊙ signifies element-wise multiplication, and PSNR and SSIM are abbreviations for Peak Signal-to-Noise Ratio and Multi-Scale Structural Similarity Index, respectively.

### 4.5 Evaluation of healthiness and identity preservation

We conducted a thorough evaluation of our proposed method alongside five other models, examining them across four essential dimensions: Healthiness (H), Mask Peak Signal-to-Noise Ratio (MPSNR), Mask Structural Similarity Index Measure (MSSIM), and Structural Healthiness (SH). The outcomes for the T1 modality are detailed in Table 1, and those for the T2 modality appear in Table 2. Under the T1 modality, the AAE model achieved the highest Healthiness score and maintained strong performance in the T2 modality. This superior performance is primarily due to the blurriness of the images it generated, which impacts the segmentor's ability to accurately locate lesion regions, thus resulting in higher health metrics. On the other hand, the PHS-GAN and GVS-GAN models, tailored specifically for brain medical imaging, significantly outshine the other models in both health and subject identity metrics. However, their heavy reliance on the segmentor for identifying lesion regions slightly compromises subject identity preservation. The AAE, VAGAN, and CycleGAN models exhibit a noticeable deficiency in preserving subject identity compared to other models, as they do not incorporate pixel-level pathological annotations, leading to less precise lesion region localization. Our method, which accurately replaces the pathological region and its adjacent 5mm area, nearly flawlessly preserves subject identity. Additionally, extensive training with healthy brain medical images allows the pseudo-healthy brain images synthesized by our method to be more coherent, ensuring a superior Healthiness score.

**Table 1:** Quantitative comparison of health and identity preservation metrics for AAE, CycleGAN, VAGAN, PHS-GAN, GVS-GAN, and the proposed method under the T1 modality.

**Models**	**Healthiness**	**MPSNR**	**MSSIM**	**Structure healthiness**
**AAE** (Makhzani et al., 2016)	**0.968**	20.64	0.795	0.702
**CycleGAN** (Zhu et al., 2020)	0.701	31.63	0.968	0.294
**VAGAN** (Baumgartner et al., 2018)	0.721	21.50	0.899	0.422
**PHS-GAN** (Xia et al., 2020)	0.831	32.18	0.987	0.580
**GVS-GAN** (Zhang et al., 2022)	0.909	33.32	0.993	0.749
**Ours**	0.929	**34.92**	**0.995**	**0.843**

Bold indicates the highest values.

**Table 2:** Quantitative comparison of health and identity preservation metrics for AAE, CycleGAN, VAGAN, PHS-GAN, GVS-GAN, and the proposed method under the T2 modality.

**Models**	**Healthiness**	**MPSNR**	**MSSIM**	**Structure healthiness**
**AAE** (Makhzani et al., 2016)	0.849	21.93	0.775	0.733
**CycleGAN** (Zhu et al., 2020)	0.744	32.98	0.964	0.496
**VAGAN** (Baumgartner et al., 2018)	0.783	22.47	0.898	0.499
**PHS-GAN** (Xia et al., 2020)	0.887	32.55	0.977	0.621
**GVS-GAN** (Zhang et al., 2022)	0.945	33.11	0.984	0.589
**Ours**	**0.957**	**33.65**	**0.992**	**0.749**

Bold indicates the highest values.

### 4.6 Evaluation of visual quality

**Figure 6:**
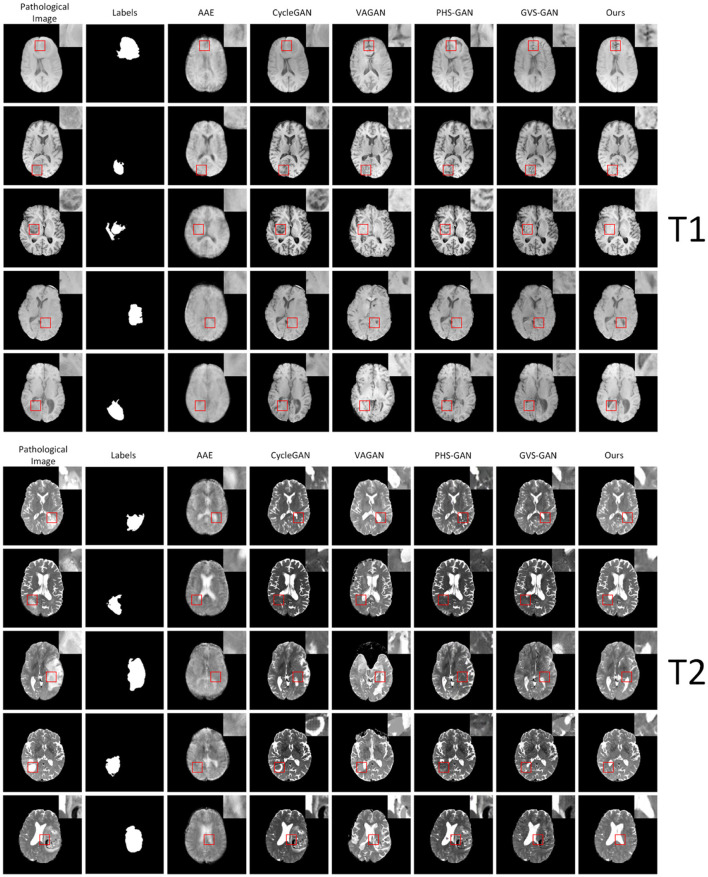
Shows experimental results on five samples (one per row) from the BraTS2021 dataset under the T1 and T2 modalities. The columns, from left to right, display the original pathological images, followed by the synthesized pseudo-healthy images generated by AAE, CycleGAN, VAGAN, PHS-GAN, GVS-GAN, and the proposed method.

We conducted experiments comparing our model against five baseline models, assessing their performance in synthesizing pseudo-healthy images under the T1 and T2 modalities, as depicted in Figure 6. Each method's efficacy was evaluated based on subject identity and healthiness. Healthiness is assessed by how well pathological and normal regions integrate in the synthesized images. Images where pathological regions blend seamlessly with normal areas are considered “healthy,” while those where pathological areas are distinctly separate are regarded as “unhealthy.” Our findings indicate that images generated by the AAE model often do not maintain the subject identity of the input images and appear notably blurred. The VAGAN-produced images can reconstruct lesion regions to a degree, but the quality of reconstruction is poor, and the inaccurate localization of lesions leads to a loss of subject identity. PHS-GAN, similar to CycleGAN, and CycleGAN itself both face challenges in preserving subject identity while repairing extensive lesion regions, resulting in some images losing subject identity and having less coherent repaired organ structures. The performance of GVS-GAN relies heavily on the segmentor's accuracy during the generation process, with errors leading to the creation of lesion-free but blurred tissue structures in the synthesized images. In contrast, our method effectively preserves subject identity in pseudo-healthy images by specifically replacing pathological regions. Enhanced by a context residual mechanism, the synthesized images exhibit a balanced tissue structure distribution, clear visual quality, and consistent preservation of subject identity.

### 4.7 Other modalities

**Figure 7:**
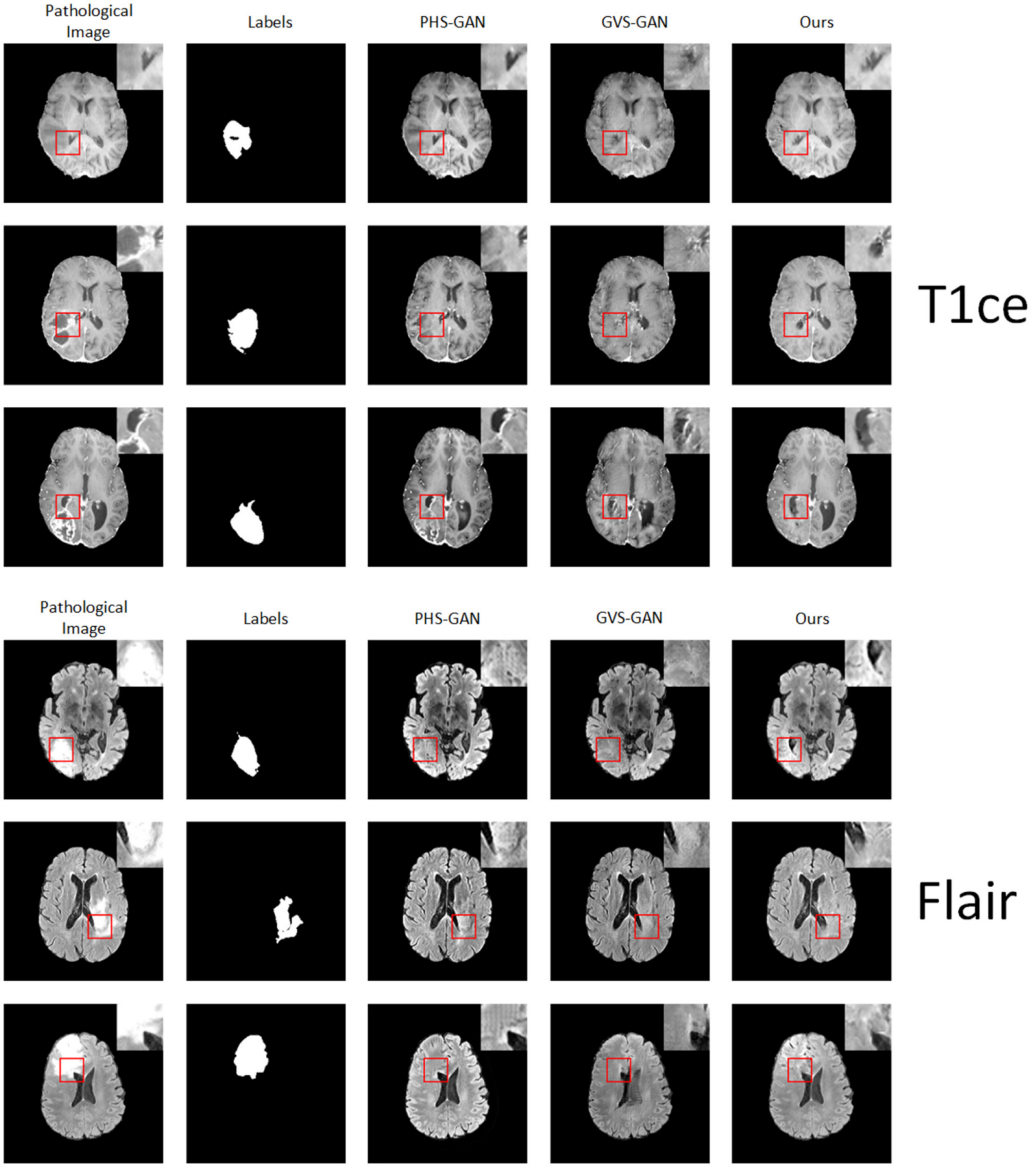
Experimental results of five samples each for T1 and T2 modalities on the BraTS dataset: original pathological images and pseudo-healthy images synthesized by PHS-GAN, GVS-GAN, and our method (from left to right).

We conducted comparative experiments focusing on the T1ce and Flair modalities, alongside the PHS-GAN and GVS-GAN models, which previously showed promising results in T1 and T2 modalities. As depicted in Figure 7, the qualitative analysis reveals that images from all three models exhibit a degree of blurring in the T1ce modality. Our model, however, demonstrates superior performance in lesion repair and restoration of brain structures compared to PHS-GAN and GVS-GAN. In the Flair modality, both PHS-GAN and our model show areas of high signal intensity, with PHS-GAN's high signal areas extending throughout the brain. Meanwhile, the images generated by GVS-GAN display no significant high signal areas but fall short in restoring brain structures effectively.

The quantitative results, as presented in Table 3, show that our approach significantly surpasses the other methods in the T1ce modality. In the Flair modality, while GVS-GAN excels in terms of healthiness, our method outperforms in other significant metrics. Overall, the qualitative and quantitative outcomes underscore our method's comparative advantage in both T1ce and Flair modalities over competing approaches, affirming its efficacy in producing more accurate and clinically relevant pseudo-healthy images.

**Table 3:** Quantitative comparison of health and identity preservation metrics for PHS-GAN, GVS-GAN, and the proposed method under the T1ce modality and flair modality.

**Modal**	**Models**	**Healthiness**	**MPSNR**	**MSSIM**	**Structure healthiness**
	**PHS-GAN** (Xia et al., 2020)	0.831	31.78	0.979	0.571
**T1ce**	**GVS-GAN** (Zhang et al., 2022)	0.909	33.13	0.989	0.529
	**Ours**	**0.935**	**37.53**	**0.994**	**0.679**
	**PHS-GAN** (Xia et al., 2020)	0.731	31.27	0.966	0.552
**FLAIR**	**GVS-GAN** (Zhang et al., 2022)	**0.912**	32.16	0.979	0.602
	**Ours**	0.891	**35.58**	**0.992**	**0.605**

Bold indicates the highest values.

### 4.8 COVID-19-20 dataset

**Figure 8:**
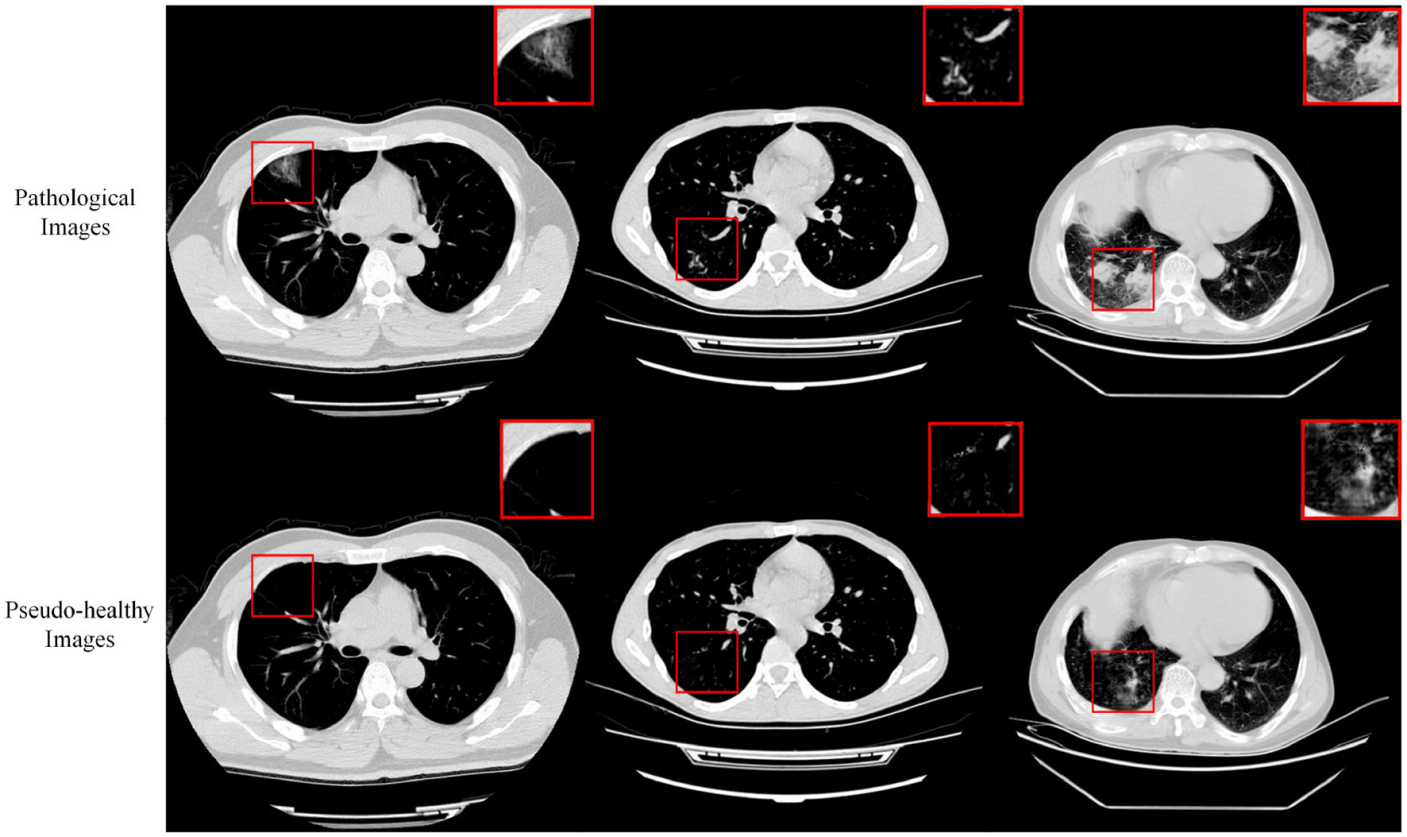
Displays the pseudo-healthy images generated by the proposed method on the COVID-19-20 dataset, with three samples shown, one per column.

Our method was applied to the COVID-19-20 dataset to generate pseudo-healthy images, specifically targeting viral lesions. Despite this, the challenges inherent in COVID-19 segmentation and the complex nature of pneumonia cases mean that pixel-level annotations are not sufficiently precise. Consequently, there is a noticeable disparity between the synthesized pseudo-healthy images and actual healthy images. As shown in Figure 8, our approach achieves some success in cases with small-scale lesions and relatively straightforward backgrounds. However, in scenarios involving extensive lung lesions, the synthesized images significantly diverge from true healthy lung images, highlighting the limitations in current segmentation and synthesis techniques in handling complex clinical scenarios.

### 4.9 Ablation study

**Figure 9:**
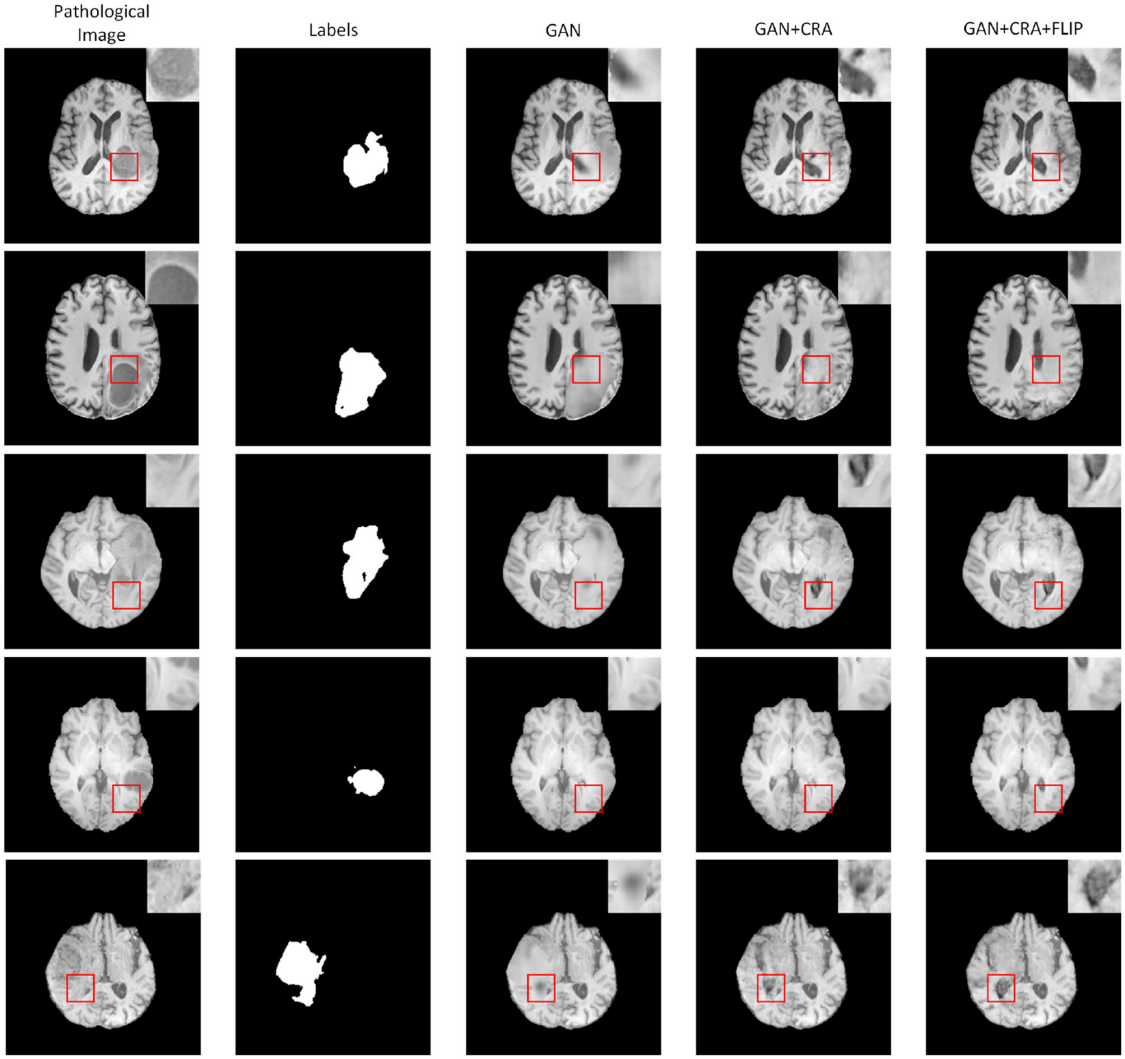
Comparison of pseudo-healthy images generated on the BraTS dataset: basic GAN, GAN with contextual residual attention, and GAN with contextual residual attention plus flipped symmetrical network across five samples (one sample per column).

To evaluate the effectiveness of the proposed flip symmetry, we conducted both qualitative and quantitative analyses on three variations of GAN networks within the T1 modality: the standard GAN, GAN with Contextual Residual Attention (GAN+CRA), and GAN with Contextual Residual Attention plus Flip Symmetry Network (GAN+CRA+FLIP). The qualitative results are illustrated in Figure 9, and the quantitative outcomes are detailed in Table 4. The findings demonstrate that networks equipped with Contextual Residual Attention significantly surpass the basic GAN in both quantitative and qualitative evaluations. Furthermore, from a qualitative standpoint, networks incorporating the flip structure produce pseudo-healthy images that exhibit greater symmetry compared to those without the flip structure. This difference in STRUCTURAL HEALTHINESS confirms that the images generated by networks with the flip structure align more closely with established health standards.

**Table 4:** Quantitative results for basic GAN networks, GAN networks with contextual residual attention mechanism, and GAN networks featuring both contextual residual attention mechanism and mirrored symmetry network.

**Models**	**Healthiness**	**MPSNR**	**MSSIM**	**Structure healthiness**
GAN	0.591	32.72	0.962	0.245
GAN + CRA	0.882	34.03	0.989	0.660
GAN + CRA + FLIP	**0.929**	**34.92**	**0.995**	**0.843**

Bold indicates the highest values.

## 5 Conclusion

We have introduced a novel pseudo-healthy synthesis method that utilizes an inpainting approach to generate images for intracranial infections. Unlike previous methods, our approach prioritizes the visual quality of the synthesized images. It consists of three components: a Segmentor, a Vague-Filler, and a Generator. The Segmentor identifies and localizes pathological regions, the Vague-Filler constructs inpainted pseudo-healthy images, and the Generator refines the reconstructions of the pathological input images. We have also established numerical evaluation metrics to assess the anatomical structure quality of the synthesized images. Demonstrated on the BraTS2021 dataset, our method exceeds current state-of-the-art benchmarks in qualitative, quantitative, and subjective evaluations.

Looking ahead, several promising research directions emerge from our work and the broader field. Our method effectively patches lesion regions, enhancing the preservation of subject identity. Post-patching, the Generator leverages global information, allowing the synthesized pseudo-healthy regions to integrate more seamlessly with adjacent areas. Although our results are impressive, our approach is limited by the need for dense, accurate segmentation annotations, which are challenging to amass in clinical settings. Future research should aim to reduce the reliance on precise pixel-level annotations, possibly through more sophisticated segmentation models or unsupervised learning techniques (Ma et al., 2024). Additionally, we have proposed a method to repair regions surrounding lesions to counteract pathologies beyond the lesion areas, though further refinement is needed for more accurate synthesis. Our method also shows limitations in synthesizing pseudo-healthy images of other organs (Liu et al., 2022), prompting future efforts to integrate more advanced localization techniques for a broader application of pseudo-healthy synthesis.

## Data Availability

The datasets presented in this study can be found in online repositories. The names of the repository/repositories and accession number(s) can be found in the article/supplementary material.
